# Genetic polymorphisms of glutathione S-transferase M1 and T1, and evaluation of oxidative stress in patients with non-small cell lung cancer

**DOI:** 10.1186/s40001-014-0067-3

**Published:** 2014-12-04

**Authors:** Hongyan Zhang, Xuwei Wu, Yi Xiao, Mei Chen, Zhidong Li, Xing Wei, Kaifa Tang

**Affiliations:** Affiliated Yan’an Hospital of Kunming Medical University, No. 245 Renmin East Road, Kunming, 650051 China; Affiliated Hospital of Guiyang Medical College, No. 9 Beijing Road, Guiyang, 550004 China

**Keywords:** Glutathione S-transferases, Polymorphism, Oxidative stress, Non-small lung cancer

## Abstract

**Background:**

Our objective is to investigate the genetic polymorphisms of the glutathione S-transferase M1 and T1 genes (GSTM1 and GSTT1) and evaluate oxidative damage in patients with non-small lung cancer (N-SCLC).

**Methods:**

One hundred and ten patients with N-SCLC and 100 controls are included in this case-control study. Multiplex polymerase chain reaction (PCR) analyses were used to identify the genotypes. The activities of malondialdehyde (MDA) and nitric oxide (NO) and total antioxidant capacity (T-AOC) were detected by spectroscopic analysis.

**Results:**

The frequencies of the GSTM1, T1, and GSTM1/T1 null genotypes in the patient group were significantly higher than that in the control group (OR = 2.071, *P* = 0.009; OR = 1.900, *P* = 0.024; OR = 3.258, *P* = 0.003). The activities of MDA and NO were significantly higher in the patient group than that in the control group (*P* <0.001), and T-AOC was significantly lower in patient group than that in control group (*P* <0.001). The activities of MDA, and NO were higher but the T-AOC was lower in patients with the GSTM1, T1 and M1/T1 null genotypes than those in patients with GSTM1, T1 and M1/T1 present genotypes (*P* <0.001).

**Conclusions:**

Our results suggest that oxidative damage may be play a important role in patients with N-SCLC, and that GSTM1 and GSTT1 null genotypes may predispose the cells of patients with N-SCLC to increased oxidative damage.

## Background

Lung cancer remains the leading cause of cancer death in the United States and in European countries, as well as in Asian countries, mainly due to late presentation [1]. Among various histological types of lung cancers, non-small cell lung cancer (N-SCLC) accounts for approximately 80%. However, knowledge on early detection, therapeutic progress, and the prognosis of N-SCLC patients remain poor [2].

Lung is a primary organ with large surface area that is directly exposed to ambient air, and therefore higher oxygen tensions, and is known to regulate reactive oxygen species (ROS) production [3]. The lung cells experience enhanced oxidative stress (OS) by exogenous free radical-generating environmental irritants and pollutants, including oxidants such as cigarette smoke, ozone, and endogenous factors like inflammation and activation of inflammatory cells [4]. Previous study has shown that oxidative stress and free radicals have been associated with an increased risk of various cancers [5]. Others studies have suggested that exposure to OS leads to single and clustered damage to cellular DNA and is involved in mutations and genomic instability, which eventually results in malignant transformation [6,7]. Ito *et a1.* demonstrated that low antioxidant capacity (AOC) in those who never smoked but that have N-SCLC may have contributed to excessive oxidative DNA damage in the lung tissues [8]. Peddireddy *et al.* showed that an evidence-based increased rate of oxidative stress plays a role in the pathogenesis of N-SCLC because a failure in the oxidant/antioxidant balance favors lipid peroxidation and DNA damage [9].

Glutathione S-transferases (GST) are a family of phase II enzymes, which uses reduced glutathione in a conjugation and in a reduction reaction to eliminate many different toxic electrophiles and products of oxidative stress [10,11]. Glutathione S-transferases (GSTM1) are able to detoxify benzopyrene diolepoxide, whereas glutathione S-transferases T1 (GSTT1) can conjugate oxidized lipids and halogenated compounds [12]. GSTM1 and GSTT1 are expressed in lung tissues [13]. GSTM1 (1p13) and GSTT1 (22q 11.2) genes, encoding for a μ-GST isoenzyme and a θ-GST isoenzyme, respectively, can be deleted, thereby causing a lack of the respective enzyme function [14]. Some previous studies suggested that GSTM1 and GSTT1 null genotypes may be associated with increased susceptibility to lung cancer [15,16], but other studies have shown that there no association between GSTM1 and T1 null genotypes for lung cancer risk [17,18].

However, few reports have investigated the relationship between GSTM1 and T1 genotypes and the level of oxidative stress (OS) in patients with N-SCLC. In this study, our intention is to determine the genotypic frequencies of the GSTM1 and T1 polymorphisms, and to evaluate the OS in patients with N-SCLC from Yunnan Province of China. The genotypes of GSTM1 and T1 and oxidative biochemical markers such as malondialdehyde (MDA), nitric oxide (NO) and total antioxidant capacity (T-AOC) were detected in each sample.

## Methods

### Participants

This case-control study consisted of 110 patients with primary N-SCLC and 100 healthy controls from Yunnan Province of China. The mean age, sex, performance status (PS), and smoking habits are shown in Table 1. N-SCLC was histologically confirmed in all patients, and all N-SCLC patients were evaluated and staged at their first visit according to medical history, physical examination including PS by Eastern Cooperative Oncology Group stage, complete blood count, serum biochemistry analyses, chest X-ray, and computed tomography scans. The controls were selected from a pool of healthy volunteers who visited the general health checkup center during the same period. A detailed questionnaire was completed for each case and control by a trained interviewer. All controls had no known medical illness or hereditary disorders and were taking no medications. The protocol was approved by the Ethics Committee of the Affiliated Yan’an Hospital of Kunming Medical University.

**Table 1:** **The descriptive statistics for the demographic and clinical characteristics of all participants**

	**N**-**SCLC** **(n =** **110)**	**Controls** **(n** = **100**)	***P*** **value**
Mean age ± SD (Age range)	60.18 ± 8.64	59.23 ± 11.12	0.493
Sex			
Female (%)	16 (14.55)	13 (13.00)	-
Male (%)	94 (85.45)	87 (87.00)	0.746
Smoking history^a^			
Mean pack-years ± SD	50.38 ± 20.25	48.02 ± 20.76	0.405
Performance status^b^			
0 to 1 (%)	83 (75.45)	-	-
2 to 4 (%)	27 (24.55)	-	-

*N*-*SCLC*, non-small cell lung cancer; *SD*, standard deviation.

^a^Smoking history in pack-years.

^b^Eastern Cooperative Oncology Group performance status.

### Glutathione S-transferase gene polymorphisms

**Figure 1:**
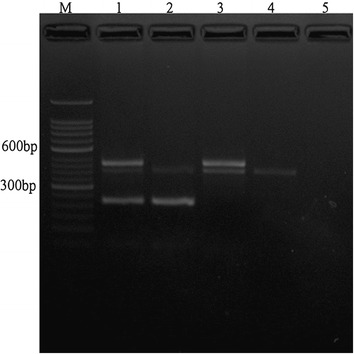
**A representative image of multiplex polymerase chain reaction (** **PCR)** **analysis of glutathione S-** **transferase M1 and T1 (GSTM1/T1),** **and β-** **actin gene polymorphisms.** Lane M, 50-bp DNA marker; Lane 1, GSTM1/T1 (+/+) genotype; Lane 2, GSTM1/T1 (+/-) genotype; Lane 3, GSTM1/T1 (-/+) genotype; Lane 4, GSTM1/T1 (-/-) genotype and Lane 5, negative control.

An AxyPrep TM Genomic DNA Miniprep Kit (Axygen Biosciences, Union City, CA, USA) was used to isolate genomic DNA from peripheral blood samples. The GSTM1 and GSTT1 genotypes were identified by multiplex polymerase chain reaction (PCR) using published primer sequences as follows: GSTM1 gene, 5′-GAA CTC CCT GAA AAG CTA AAG C-3′ (forward) and 5′-GTT GGG CTC AAA TAT ACG GTG G-3′ (reverse); GSTT1 gene, 5′-TTC CTT ACT GGT CCT CAC ATC TC-3′ (forward) and 5′-TCA CCG GAT CAT GGC CAG CA-3′ (reverse); and a 400-bp fragment for the β-actin gene 5′-ACT CCC CAT CCC AAG ACC-3′ (forward) and 5’-CCT TAA TGT CAC GCA CGA T-3’ (reverse) was used as an internal control for DNA amplification [19] (Figure 1).

### Measurement of malondialdehyde, total antioxidant capability and nitric oxide

The contents of malondialdehyde (MDA), total antioxidant capability (T-AOC) and nitric oxide (NO) in plasma were assayed using colorimetric methods with a spectrophotometer (Biomate 5, Thermo Electron Corporation, Rochester, NY, USA). The assays were conducted using the assay kits purchased from Nanjing Jiancheng Institute of Bioengineering (Nanjing, China) and used according to the manufacturer’s instructions.

### Statistical analysis

The χ^2^ test was used to compare sex and cigarette smoke between patients and controls. All data are expressed as the mean ± standard deviation (SD). Age, smoking index expressed as pack-years (number of cigarettes smoked per day × number of years smoked/20) were compared using the unpaired Student’s *t* test. The differences in the frequencies of the GST genotypes between groups were analyzed using the χ^2^ test, and the odds ratios (OR) with 95% confidence intervals (CI) are reported. The difference between the two groups was analyzed by unpaired *t* test with two-tailed values, and *P* <0.05 was considered statistically significant. All analyses were performed using SPSS software version 16.0 (SPSS, Inc., Chicago, IL, USA).

## Results and discussion

No statistically significant differences were found between patients and controls with respect to age, sex and smoking history. The frequencies of the GSTM1 and T1 genotypes in cases and controls are shown in Table 2. The frequency of the GSTM1 null genotype was 42.0% in the control group and 60.0% in the patient group (OR = 2.071; CI 95%, 1.194 to 3.593; *P* = 0.009), and 34.0% and 78.0% (OR = 4.132; CI 95%, 1.957 to 8.724; *P* <0.001) in only GSTT1 null genotype cases, and 51.1% and 52.9% (OR = 0.719; CI 95%, 0.298 to 1.734; *P* = 0.462) in only GSTT1 present genotype cases. The frequency of the GSTT1 null genotype was 53.0% in the control group and 69.2% in the patient group (OR = 1.900; CI 95%, 1.084 to 3.331; *P* = 0.024), and 52.9% and 77.3% (OR = 4.533; CI 95%, 1.958 to 10.496; *P* <0.001) in only GSTM1 null genotype cases, and 60.3% and 54.5% (OR = 1.826; CI 95%, 0.826 to 4.036; *P* = 0.135) in only GSTM1 present genotype cases. The frequency of the GSTM1/T1 null genotype was 18.0% in the control group and 46.4% in the patient (OR = 3.258; CI 95%, 1.457 to 7.287; *P* = 0.003). There were significant differences between the control group and patient group with respect to the frequencies of the GSTM1 and T1 genotypes in this study.

**Table 2:** **The distribution of glutathione S**-**transferase (GST) M1 and T1 genotypes in study groups**

**Group**	**N**-**SCLC (%) (** **n =** **110)**	**Control (%) (** **n =** **100)**	**χ** ^**2**^	***P*** **value**	**OR (** **CI 95%)**
GSTT1 total	(n = 110)	(n = 100)			
GSTM1(+)	44 (40.0)	58 (58.0)	-	-	-
(-)	66 (60.0)	42 (42.0)	6.794	0.009	2.071 (1.194 to 3.593)
GSTT1(-)	(n = 75)	(n = 53)			
GSTM1(+)	24 (32.0)	35 (66.0)	-	-	-
(-)	51 (78.0)	18 (34.0)	14.480	<0.001	4.132 (1.957 to 8.724)
GSTT1(+)	(n = 35)	(n = 47)			
GSTM1(+)	20 (57.1)	23 (48.9)	-	-	-
(-)	15 (52.9)	24 (51.1)	0.542	0.462	0.719 (0.298 to 1.734)
GSTM1 total	(n = 110)	(n = 100)			
GSTT1(+)	35 (31.8)	47 (47.0)	-	-	-
(-)	75 (69.2)	53 (53.0)	5.073	0.024	1.900 (1.084-3.331)
GSTM1(-)	(n = 66)	(n = 42)			
GSTT1(+)	15 (22.7)	24 (57.1)	-	-	-
(-)	51 (77.3)	18 (52.9)	13.177	<0.001	4.533 (1.958-10.496)
GSTM1(+)	(n = 44)	(n = 58)			
GSTT1(+)	20 (45.5)	23 (39.7)	-	-	-
(-)	24 (54.5)	35 (60.3)	2.233	0.135	1.826 (0.826-4.036)
GSTM1/T1					
(+/+)	20 (18.2)	23 (23.0)	-	-	-
(+/-)	24 (21.8)	35 (35.0)	0.345	0.557	0.789 (0.357-1.743)
(-/+)	15 (13.6)	24 (24.0)	0.542	0.462	0.719 (0.298-1.734)
(-/-)	51 (46.4)	18 (18.0)	8.571	0.003	3.258 (1.457-7.287)

+, present genotype; -, null genotype; OR, odds ratio; CI, confidence intervals.

Glutathione S-transferase M1 and T1 polymorphisms with respect to MDA, NO, and T-AOC in the plasma are displayed in Table 3. The data of MDA, NO and T-AOC were distributed in a nearly normal fashion. The activity of MDA and NO were significantly higher in the patient group than in the control group (*P* <0.001), and T-AOC was significantly lower in the patient group than in the control group (*P* <0.001). Furthermore, the activities of MDA and NO in the GSTM1, T1 and M1/T1 null genotype groups were statistically significantly higher than that with the GSTM1, T1 and M1/T1 present genotypes (*P* <0.001). However, the levels of T-AOC in the GSTM1, T1 and M1/T1 null genotypes groups were statistically significantly lower than that with the GSTM1, T1 and M1/T1 present genotypes (*P* <0.001). In addition, we found that the GSTM1 and GSTT1 genotypes also affect the levels of MDA, NO and T-AOC in the control group. The levels of MDA and NO in the GSTM1 and GSTT1 null genotype groups were higher than that in present genotype group, and T-AOC was lower in present genotype group (*P* <0.05).

**Table 3:** **Genetic polymorphisms of glutathione S**-**transferase (GST) M1 and T1 in relation to plasma MDA**, **NO and T**-**AOC**

**Group**	**N**	**MDA (** **nmo/l** **mL)**					**NO (** **nmol/** **mL)**					**T-** **AOC (** **units**/**mL)**				
Control	100	8.49 ± 3.30					16.79 ± 5.37					13.69 ± 4.56				
Case	110	11.67 ± 4.31					19.68 ± 4.11					9.88 ± 3.58				
*P*		<0.001					<0.001					<0.001				
GSTM1																
(+)	44	10.18 ± 4.23					18.01 ± 3.56					11.11 ± 2.67				
(-)	66	12.67 ± 4.09					20.80 ± 4.09					9.07 ± 3.88				
*P*		<0.001^a^; 0.003^b^					<0.001^a^; <0.001^b^					<0.001^a^; 0.001^b^				
GSTT1																
(+)	35	9.29 ± 2.84					17.98 ± 3.99					11.73 ± 2.82				
(-)	75	12.78 ± 4.44					20.48 ± 3.94					9.03 ± 3.58				
*P*		<0.001^a^; <0.001^c^					<0.001^a^; 0.003^c^					<0.001^a^; <0.001^c^				
GSTM1/T1		Control		Case		*P*	Control		Case		*P*	Control		Case		*P*
(+/+)		23	7.81 ± 2.97	20	8.56 ± 2.86	0.047	23	15.74 ± 4.38	20	17.68 ± 3.57	0.049	23	14.71 ± 4.29	23	11.97 ± 2.28	0.046
(+/-)		35	8.45 ± 3.01	24	11.52 ± 4.76	0.018	35	16.05 ± 5.13	24	18.28 ± 3.60	0.032	35	13.68 ± 4.79	35	10.39 ± 2.80	0.028
(-/+)		24	8.47 ± 2.98	15	10.27 ± 2.60	0.021	24	16.23 ± 4.90	15	18.37 ± 4.59	0.027	24	13.59 ± 4.92	24	11.40 ± 3.47	0.024
(-/-)		18	8.81 ± 3.12	51	13.38 ± 4.20	0.008	18	18.02 ± 4.29	51	21.51 ± 3.68	0.004	18	10.27 ± 5.01	18	8.39 ± 3.75	0.003
*P*				<0.001^a^; <0.001^d^					<0.001^a^; <0.001^d^					<0.001^a^; <0.001^d^		

+, present genotype; -, null genotype; MDA, malondialdehyde; NO, nitric oxide; T*-*AOC, total antioxidant capacity.

^a^compared with control.

^b^compared with GSTM1 (+).

^c^compared with GSTT1 (+).

^d^compared with GSTM1/T1 (+/+).

Oxidative stress (OS) is defined as an imbalance between production of free radicals and reactive metabolites, which are called reactive oxygen species (ROS), and ROS elimination by protective mechanisms which are referred to as antioxidants, and this imbalance leads to damage of important molecules and cells, with potential impact on the whole organism [20]. In addition, cancer initiation and progression have been shown to be associated with oxidative stress by increasing DNA mutations or inducing DNA damage, genome instability, and cell proliferation [21]. Previous studies showed that an increased rate of oxidative stress plays an important role in the pathogenesis of N-SCLC [8,9]. In the current study, our results suggest that the levels of MDA and NO were higher in the patient group than that in the control group, but the level of T-AOC was lower in the patient group than that in the control group.

Glutathione S-transferases (GSTs), an important super family of phase II drug-metabolizing enzymes that respond to oxidative stress, include at least seven distinct classes, namely, α (A), μ (M), π (P), σ(Sigma), ζ (Zeta), ω (Omega) and θ (T), and play an important role in cell protection by catalyzing the conjugation of a large variety of endogenous and exogenous compounds, including ROS and carcinogenic compounds and their metabolites. Human cytosolic GST genes exhibit genetic polymorphisms, and many genetic polymorphisms lead to altered GST activities, which may be partially responsible for the individual host’s susceptibility to oxidative damage. Previous studies suggested that GSTM1 and GSTT1 null genotypes may be associated with increased susceptibility to lung cancer [15,16], but other studies have shown no associations between GSTM1 and T1 null genotypes for lung cancer risk [17,18]. In our results, we found that the frequencies of the GSTM1, T1, and M1/T1 null genotypes were significantly higher in the patient group than that in the control group. Moreover, we found that OR for GSTM1 (null/present) in T1 null genotype (OR = 4,132) and present genotype (OR = 0,719) groups, and OR for GSTT1 (null/present) in M1 null genotype (OR = 4.533) and present genotype (OR = 1.826) groups are heterogeneous. It was shown that a significant susceptibility to oxidative damage is not attributable to the presence of either the M1 or T1 gene. Both genes must be positive for the significance of OR.

Furthermore, the association between the GSTM1 and T1 genotypes and level of oxidative stress in the patient group was analyzed in the current study. We found that the activities of MDA and NO in the GSTM1, T1 and M1/T1 null genotype groups were statistically significantly higher than that in the GSTM1, T1 and M1/T1 present genotype groups. The level of T-AOC in the GSTM1, GSTT1 and GSTM1/T1 null genotype groups were statistically significantly lower than that in the GSTM1, T1 and M1/T1 present genotype groups. Moreover, we found that for one of the genes of M1 and T1 to be positive is not sufficient for susceptibility to oxidative damages. Both genes must be positive for an important increase in susceptibility to oxidative damages.

## Conclusions

Our results suggest that oxidative damage may play an important role in patients with N-SCLC and that the GSTM1 and GSTT1 null genotypes may predispose the tissues of patients with N-SCLC to increased oxidative damage. Both null genotypes must be positive for an important increase in the susceptibility to oxidative damages. Therefore, more attention should be paid to oxidative stress-related pathological manifestations in patients with N-SCLC who bear the GSTM1 and GSTT1 null genotypes. Furthermore, studies on glutathione S-transferases gene polymorphisms and levels of oxidative stress should be done in a multicenter, multi-ethnic population and with a large number of patients with N-SCLC in the future.

## Abbreviations

GSTM1: glutathione S-transferase M1
GSTT1: glutathione S-transferase T1
N-SCLC: non-small lung cancer
PCR: multiplex polymerase chain reaction
OS: oxidative stress
MDA: malondialdehyde
NO: nitric oxide
T-AOC: total antioxidant capacity
